# In Vitro Investigation of Perfluorooctane Sulphonate and Perfluorooctanoic Acid on Boar Spermatozoa Agglutination

**DOI:** 10.1002/vms3.70244

**Published:** 2025-03-17

**Authors:** Paola Berenice Ortiz‐Sánchez, Yolitzin A. Miranda‐Ruiz, Leslye Sámano‐Hernández, Irma Jiménez‐Morales, Humberto González‐Márquez, Reyna Fierro

**Affiliations:** ^1^ División de Ciencias Biológicas y de la Salud, Departamento de Ciencias de la Salud Universidad Autónoma Metropolitana ‐ Iztapalapa CDMX Mexico

**Keywords:** agglutination, boar spermatozoa, capacitation, d‐penicillamine, perfluorooctane sulphonate, perfluorooctanoic acid, rosette

## Abstract

Various toxic agents are associated with male infertility, including perfluoroalkyl substances (PFAS), which are emerging as significant contributors due to their physico‐chemical properties that exhibit a propensity for bioaccumulation and potentially pose reproductive risks. The aim of this study was to analyse the effects of perfluorooctane sulphonate (PFOS) and perfluorooctanoic acid (PFOA) on boar spermatozoa during capacitation through a focus on agglutinate formation. A second objective was to assess the influence of d‐penicillamine (d‐Pen) on boar spermatozoa agglutinate prevention. PFOS and PFOA in sublethal concentrations reduced the proportion of spermatozoa that achieved capacitation, PFOS increased to 35% the spermatozoa mortality and both toxic compounds generated an abnormally excessive increase in rosette‐type agglutinates that formed non‐mobile aggregates (only vibrant), like those that form during physiological capacitation. This increase in agglutinates came at the expense of available free spermatozoa for fertilisation. Treatment with d‐Pen, however, significantly reduced rosette formation by PFAS. Further study of the underlying mechanisms revealed that exposure to PFOS and PFOA led to decreased free sulfhydryl (SH) groups on the surface of the spermatozoa, likely due to oxidation caused by the PFAS. Administering d‐Pen also reversed this effect, suggesting a possible involvement of S–S bond formation during spermatozoa agglutination. These findings not only shed light on how PFOS and PFOA affect boar spermatozoa capacitation, but also shows the mechanism associated with spermatozoa rosette‐type agglutination provoked by PFOS and PFOA. Furthermore, they underscore the need to delve more deeply into the mechanisms that govern agglutinate formation during spermatozoa capacitation physiology to devise improved, targeted therapeutic strategies for male infertility and enhance animal reproduction.

## Graphical abstract

•PFOS and PFOA induce rosettes‐like agglutination through SH oxidation and disulphide bond formation, leading to immobile sperm clusters.

•d‐Penicillamine, a drug already used without side effects, prevents this PFAS agglutination by inhibiting oxidative processes.

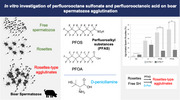

## 1 Introduction

Spermatozoa agglutination is a phenomenon that often causes male infertility. Since the release of spermatozoa from their conjugates is a fundamental requirement for fertilising the egg (Monclus and Fornes 2016). Although anti‐spermatozoa antibodies (ASAs) (Chereshnev et al. 2021) have been identified as a common cause of spermatozoa agglutination, other etiologies, such as heavy metals, bacteria and nutritional deficiencies, have also been implicated in their appearance (Berger et al. 2019). Among these triggering factors, the bicarbonate ion, calcium and bovine serum albumin (BSA) stand out as they have been associated with the formation of spermatozoa agglutination in males such as boars and horses (Harayama et al. 1998; Leemans et al. 2016). Sperm–sperm association can occur in various ways, including head–head, head–flagellum and flagellum–flagellum. Monclus and Fornes (2016) highlighted the complexity of spermatozoa interaction and noted that several terms have been used to describe these interactions: conjugates, complexes, aggregates, associates, rouleaux or rosettes. This variety of terms has generated great confusion, underscoring the need to homogenise a nomenclature that accurately defines the characteristics of each form. For example, rosettes are distinguished by having only head–head interactions, while aggregates are formed mainly due to xenobiotics in the environment where the spermatozoa are located that generate nonspecific interactions (Monclus and Fornes 2016).

The capacitation process is crucial because it triggers fundamental changes that give spermatozoa their fertilising capacity. In boars and guinea pigs, rosettes consist of small groups of spermatozoa interconnected in a specific head–head manner. These physiological rosettes do not impede fertilisation, as they have been associated with efficient capacitation in the oviductal reservoir (Harayama et al. 1998; Salgado‐Lucio et al. 2020). Agglutinates have been observed from the epididymis and predominate during capacitation, especially in the oviductal reservoir of females, where the union of spermatozoa to the oviductal epithelial cells takes place through carbohydrate residues and lectins (Flaherty et al. 1993; Harrison 1997; Hiroshi and Seishiro 2001; Kolle 2022). However, questions persist regarding the contribution of these agglutinates to spermatozoa fertility, the mechanisms that underlie their formation, and the possible effects of xenobiotics.

Leahy et al. (2016) have shown that copper can form agglutinates in ram spermatozoa without affecting motility. These agglutinates are generated when cupric ions oxidise sulfhydryl radicals (RSH) to form a Cu^2+^‐RSH complex, in which Cu^2+^ is reduced to Cu^+^ and RSH is oxidised to RS•. This allows the formation of disulphide bonds between spermatozoa by employing S–S. Subsequently, it was found that the reducing agent d‐penicillamine (d‐Pen) can prevent or reverse this agglutination because it reduces the disulphide bonds of a copper‐binding protein in spermatozoa (Leahy et al. 2016). This mechanism of Cu^2+^ agglutination through the oxidation of sulfhydryl (SH) groups could act in a similar manner to other oxidising compounds, such as perfluoroalkyl substances (PFAS). Evaluating whether this mechanism is analogous to that proposed by Leahy's group is a key topic of interest in the present study.

Exposure to persistent environmental pollutants, such as heavy metals, pesticides or PFAS, raises questions about their influence on mammalian reproduction. PFAS are chemically synthesised perfluorinated carbon chains with an amphiphilic character. Their alkyl chain is hydrophobic, and they have a hydrophilic functional group that gives them notable chemical and thermal stability and high surface activity. Among the PFAS, perfluorooctanoic acid (PFOA) and perfluorooctane sulphonate (PFOS) have been widely used in various industries since the 1940s in numerous everyday products like textiles and food packaging (Environmental Protection Agency (EPA) 2021; Olsen et al. 2007). PFOS and PFOA have been identified in human blood serum. Concentrations of 131 ng/mL of PFOS and 17.7 ng/mL of PFOA have been documented (Kato et al. 2011). PFOS and PFOA have half‐lives of 4.8–5.4 and 3.5–3.8 years, respectively (Olsen et al. 2007).

Both compounds have been associated with adverse effects on male fertility, especially decreased spermatozoa quality and reduced spermatogenesis. They are considered endocrine disruptors due to their ability to alter the functionality of the hypothalamus–pituitary–gonad axis (Rickard et al. 2022). In recent studies, PFOS and PFOA were found in the follicular fluid of women who were undergoing fertility treatments at concentrations of 0.7–22.4 and 2.4–14.5 ng/mL, respectively. The presence of PFAS in follicular fluid was not directly related to a change in fertilisation rates. It was associated with certain factors involved in female infertility, such as endometriosis, polycystic ovary syndrome, genital tract infections and idiopathic factors (Donley et al. 2019; Kim et al. 2020).

Workers exposed occupationally to PFOA and adult rats, who were treated with this substance, showed increased estradiol levels and low testosterone production (Cook et al. 1992; Gilliland and Mandel 1993). Likewise, high concentrations of PFOS have been associated with reduced testosterone levels (Joensen et al. 2013). Other studies in occupationally exposed workers in China have reported serum levels of 118,000 ng/mL (235.94 µM) for PFOS and 32,000 ng/mL (77.28 µM) for PFOA (França et al. 2005; Fu et al. 2016). Farm animals, such as boars, may be directly at risk of exposure to PFAS through a variety of environmental pathways and sources; the primary route of exposure is diet, including ingesting contaminated water; farm animals grazing on contaminated soil may also be exposed through dust and air particles contaminated by industrial products or waste used in the field, such as pesticides. The highest PFAS concentrations in wildlife tend to be associated with proximity to contaminated sites (De Silva et al. 2021).

PFOS and PFOA have been shown to cause dysfunction in the plasma membrane, leading to a decrease in the proportion of spermatozoa that achieved capacitation and the acrosomal reaction, together with damages such as deregulation of the membrane potential, accumulation of intracellular calcium levels, inhibition of cholesterol efflux and failures in the correct rearrangement of membrane components during capacitation (Ortiz‐Sanchez et al. 2022). Therefore, the present study was designed to examine the degree of agglutination induced by PFOS and PFOA during capacitation and its possible relation to disulphide bond formation (S–S) in membrane proteins. This research could improve the understanding of the mechanisms that underlie boar spermatozoa agglutination induced by perfluoroalkylated compounds, opening new perspectives on potential strategies to mitigate their adverse effects, specifically in the formation of agglutinates in mammals and improving the animal reproduction programs.

## 2 Materials and Methods

Unless otherwise specified, all chemicals were purchased from Sigma Chemical Company (St. Louis, MO). Optical and epifluorescence microscopes (ZEISS, Germany) with 100 and 400× magnifications were used. All analyses were conducted using the Axio Vision by Zeiss Zen software (USA). At least 200 spermatozoa were evaluated per sample and in duplicate, totalling five independent samples.

### 2.1 Experimental Design

**FIGURE 1:**
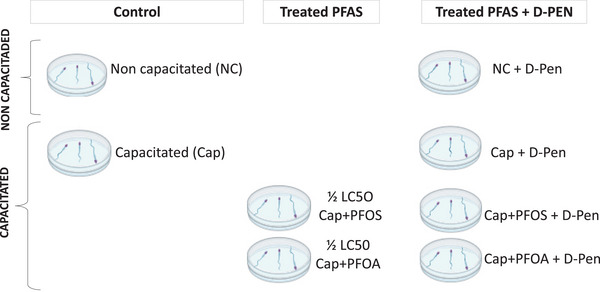
Experimental design to agglutination spermatozoa analysis. Two principal groups (non‐capacitated and capacitated) are divided in three experimental: Controls, treated PFAS (½ LC50) and treated PFAS + D‐Pen. NC, non‐capacitated; Cap, capacitated. PFOS (½ LC50 = 230 µM); PFOA (½ LC50 = 947 µM); D‐Pen (1 mM).

First, two control groups were used to analyse large aggregates induced by PFOS and PFOA: Non‐capacitated (NC) and capacitated (Cap) spermatozoa without PFAS; the concentrations of PFAS in this section were 1 and 1.5 mM. For the analysis of spermatozoa agglutination at sublethal concentrations, two control groups were established: NC spermatozoa; Cap spermatozoa. In addition, two Cap groups treated with PFAS were formed: Cap + PFOS and Cap + PFOA. All these groups + d‐Pen were included in the analyses. In previous works, the LC50 of PFOS and PFOA for Cap spermatozoa of boar was determined (460 µM to PFOA and 1894 µM to PFOA) (Ortiz‐Sanchez et al. 2022; Oseguera‐Lopez et al. 2020). The concentrations of PFAS used in this section were ½ LC50 of PFOS (230 µM), ½ LC50 of PFOA (947 µM) and d‐Pen (1 mM). Concentrations of PFAS are in the same order of magnitude as those found in some occupationally exposed personnel (França et al. 2005; Fu et al. 2016). The concentration of d‐Pen was the same as that used by the authors Leahy et al. (2016), in which copper agglutination was prevented by d‐Pen (Figure 1).

### 2.2 Semen Samples

Five healthy, fertile Landrace boars of reproductive age (1–4 years) were used. Semen samples were obtained using the glove‐hand method from a commercial boar stud, which complies with the health and animal welfare regulations of the government of Mexico, according to the Official Standard NOM‐062‐ZOO‐1999, which stipulates the regulations for the care and use of laboratory animals.

A high‐quality sample was considered when the following criteria were met: viability and total motility > 80%, abnormalities < 15% and a concentration > 2 × 10^8^ spermatozoa/mL. Mortality was determined using the Eosin/Nigrosin (E–N) staining technique, where 5 µL of a spermatozoa sample was mixed with 5 µL of E–N, and a smear was made on slides and analysed under bright field microscopy. The ones that stained pink were considered dead. The proportion of spermatozoa with morphological abnormalities was also assessed from the same smears. Total motility was analysed using optical microscopy by directly observing and assigning a motility percentage to different observed fields. The concentration was determined by counting in a Neubauer chamber. All these parameters were studied in 200 spermatozoa per sample and in duplicate. This quality evaluation was carried out to select only normozoospermic samples, following previously established criteria (Alkmin et al. 2013; Jimenez et al. 2003).

### 2.3 Induction of Spermatozoa Capacitation

To remove seminal plasma, the samples were washed twice with phosphate‐buffered saline (PBS) and centrifuged at 600 × *g* for 5 min. After that, 5 × 10^6^ spermatozoa in 1000 µL of medium were placed in a four‐well culture plate. For NC, it was used HEPES‐TALP medium (KCl 3.1 mM, NaCl 100 mM, NaH_2_PO_4_·H_2_O 0.29 mM, Hepes 10 mM, NaHCO_3_ 2.5 mM, sodium lactate 21.6 mM, CaCl_2_·2H_2_O 2.1 mM and MgCl_2_·6H_2_O 1.5 mM) at pH 7.4 corresponding to unsupplemented non‐capacitating medium (NCM). For the Cap group, the medium HEPES‐TALP was supplemented with 6 mg/mL BSA fraction V and 1 mM sodium pyruvate. The spermatozoa were incubated for 4 h at 38°C in a humidified atmosphere with 5% (v/v) CO_2_. Capacitation was assessed by chlortetracycline (CTC) staining. In each case, the spermatozoa were fixed for 1 h with 5 µL of 0.2% glutaraldehyde in 0.5 M Tris buffer, pH 7.4, then 5 µL of spermatozoa were mixed with 5 µL of 750 µM CTC buffer. Samples were mounted on microscope slides with Gelvatol and covered with glass coverslips. Slides were observed under 495 nm UV epifluorescence. Only the samples containing over 60% of Cap spermatozoa were used. CTC fluorescence patterns were determined as follows: NC fluorescence throughout the spermatozoa head; Cap intense fluorescence in the equatorial and acrosomal zone; and acrosomal‐reacted, AR, fluorescence in the equatorial and, in some cases, the post‐equatorial zone (Jimenez et al. 2006; Ortiz‐Sanchez et al. 2022; Ward and Storey 1984).

### 2.4 Mortality of PFAS During Capacitation

The E–N staining technique was used to evaluate the mortality effect of PFOS and PFOA sublethal concentrations during capacitation. Stained spermatozoa were considered dead. The cytotoxicity of the diluents of both toxicants, DMSO for PFOS and NCM for PFOA, was also evaluated as a negative control.

### 2.5 Degrees of Agglutination

**FIGURE 2:**
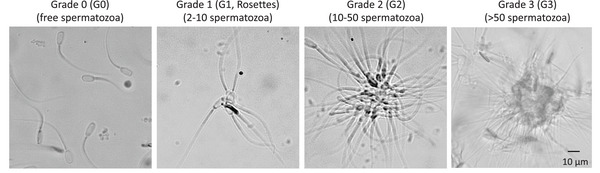
Agglutination grades were found in this work. G0: free spermatozoa non‐bounded, G1: rosettes joined head–head (2–10 spermatozoa); G2: head‐head (10–50 spermatozoa); G3: > 50 spermatozoa, considered aggregates.

We determined the type and grade of agglutination present. Findings showed four grades, as shown in Figure 2, concurred with the degrees reported in the WHO's laboratory manual for semen examination and processing (World Health Organization 2021). These grades are classified as follows: G0 corresponds to free spermatozoa; G1 shows rosettes that have 2–10 spermatozoa joined head–head; G2 refers to a group of 10–50 spermatozoa joined head–head; while (G3) indicates over 50 spermatozoa that are joined together (Figure 2).

### 2.6 Agglutination Analysis

An aliquot of 30 µL was taken from each group for microscopic observation in a random field; 200 agglutinates per group were counted. We determined the type and grade of agglutination present. Findings showed four grades, as shown in Figure 2, concurred with the degrees reported in the WHO's laboratory manual for semen examination and processing (World Health Organization 2021). These grades are classified as follows: G0 corresponds to free spermatozoa; G1 shows rosettes that have 2–10 spermatozoa joined head–head; G2 refers to a group of 10–50 spermatozoa joined head–head; while (G3) indicates over 50 spermatozoa that are joined together. This procedure was repeated to obtain *n* = 5 in each case.

### 2.7 Evaluation of the Free SH Groups

The presence of free SH groups on the surface of the spermatozoa was evaluated to determine if the effect of d‐Pen on the decrease in agglutinated rosettes is coupled with a reduction of disulphide bonds. SH groups were determined using the reagent 5‐Iodoacetamide fluorescein (5‐IAF). After capacitation, the spermatozoa were fixed, as mentioned above. The sample was incubated at a final concentration of 0.1 mM 5‐IAF for 15 min at 37°C in darkness. Subsequently, washing was performed by centrifugation for 5 min at 600 × *g*, followed by adding PBS. A 10µL aliquot was placed on a slide, and the slides were analysed under fluorescence microscopy, counting at least 200 cells. Head spermatozoa fluorescence was categorised into three patterns: low (slight fluorescence), intermediate and intense (bright fluorescence) (Alhathal et al. 2016). ImageJ software quantified fluorescence intensity using arbitrary fluorescence units (A.F.U.). Three levels were arbitrarily chosen to categorise fluorescence. The first corresponds to low fluorescence, the second to intermediate fluorescence and the last to intense fluorescence. The intermediate and intense fluorescence values were considered indicators of the presence of SH groups.

### 2.8 Statistical Analysis

The mean and standard deviation of each capacitation and mortality analysis, agglutinate classification and SH group were obtained by analysing at least 200 agglutinates. An ANOVA and Tukey post‐hoc tests were done. A value of *p* < 0.05 was a significant difference. All calculations were made with SPSS software (IBM SPSS Statistics for MacOS, Version 20.0, Armonk, NY: IBM Corp).

## 3 Results

### 3.1 Sublethal Concentrations of PFOS and PFOA Decrease Spermatozoa Capacitation

**FIGURE 3:**
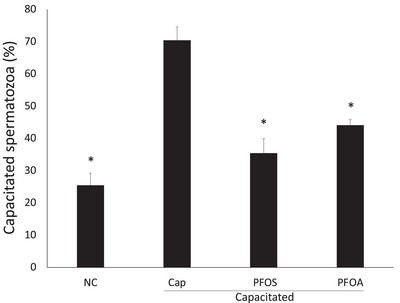
Effect of PFOS and PFOA on capacitation process. Sublethal concentrations of PFOS and PFOA reduced the proportion of capacitated spermatozoa. NC, non‐capacitated; Cap, control capacitated; PFOS = 230 µM and PFOA 947 µM (½ LC50). Stain: CTC capacitated pattern. Mean ± SD, *p* < 0.05. *Significant differences compared to group Cap; *n* = 5 in duplicate.

To corroborate the effects of PFOS and PFOA on spermatozoa capacitation, sublethal doses of ½ LC50 of toxicants (230 µM of PFOS, 947 µM of PFOA) were used. The spermatozoa were incubated in a suitable medium to induce capacitation HEPES‐TALP. The Cap control group achieved 70% capacitation. However, PFOS and PFOA significantly decreased capacitation to 35% and 44%, respectively (Figure 3).

### 3.2 The Sublethal Concentration of PFOS Increases the Mortality of Spermatozoa During Capacitation

**FIGURE 4:**
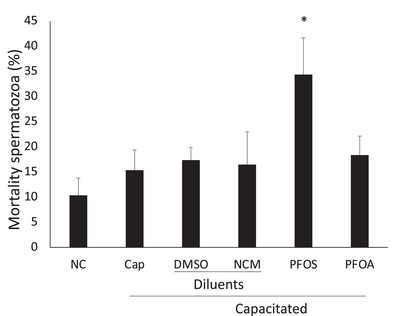
Effect of PFOS and PFOA on capacitated sperm mortality. Sublethal concentration of PFOS increases the proportion of dead capacitated spermatozoa. No significant differences appeared with PFOA NC: non‐capacitated; Cap: control capacitated; PFOS = 230 µM and PFOA 947 µM (½ LC50). Stain: Eosin–Nigrosin. Mean ± SD, *p* < 0.05. *Significant differences; *n* = 5 in duplicate.

After capacitation, the proportion of dead spermatozoa, as the toxic effect, was determined. As a negative control, the spermatozoa cytotoxicity of the PFAS diluents (DMSO for PFOS, NCM for PFOA; different diluents were used because solubility is dissimilar). No cytotoxic effect was observed with the PFAS diluents, so they are not mentioned in the description of the subsequent experiments. The Cap control group achieved only 15% of mortality. However, PFOS significantly increased mortality to 34%. No significant differences appeared with PFOA (Figure 4).

### 3.3 PFOS and PFOA at High Concentrations Induce Large, Non‐Mobile Progressive Aggregate‐Type Agglutinates During Capacitation

**FIGURE 5:**
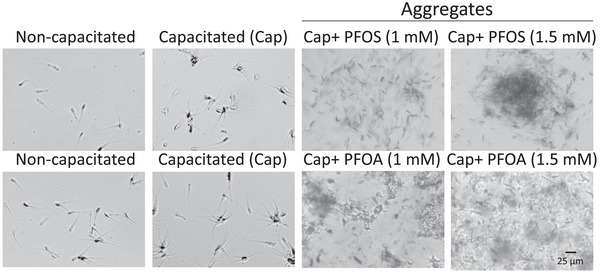
Effect of PFOS and PFOA at high concentrations on spermatozoa agglutination. Large spermatozoa aggregates formed in the presence of 1 and 1.5 mM PFOS and PFOA. The aggregates do not have progressive mobility, only vibrating. Control groups: Non‐capacitated corresponding for free spermatozoa and capacitated (Cap) for rosettes type G1. *n* = 5 in duplicate.

Inducing the capacitation of boar spermatozoa with high concentrations of PFOS and PFOA significantly increased spermatozoa agglutination compared to the NC and Cap control groups. In Figure 1, at 1 mM of PFOS and 1.5 mM of PFOA, spermatozoa aggregates were larger and had no spaces between the cells in most observed fields, which made quantifying them quite difficult. These aggregates were non‐mobile progressive, with only vibrant mobility at 1 mM and non‐mobile at 1.5 mM. Vibrating type mobility corresponds to the few spermatozoa alive at 1 mM. The compounds PFOS and PFOA produce notable effects on agglutination (Figure 5).

### 3.4 Sublethal Concentrations of PFOS and PFOA Induced an Increment of Small Rosettes During Spermatozoa Capacitation

**FIGURE 6:**
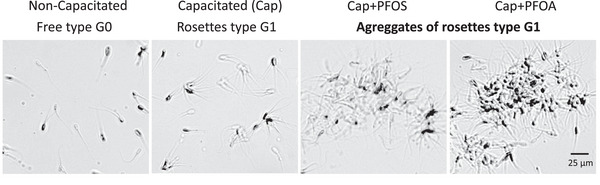
Effect of PFOS and PFOA at sublethal concentrations on spermatozoa agglutination. PFOS and PFOA increased the number of non‐mobile, aggregates of rosettes agglutinates (G1) during capacitation. Non‐capacitated: free spermatozoa (G0); capacitated (Cap): G1 rosettes; C + PFOS and C + PFOA form aggregates of rosettes type G1; *n* = 5 in duplicate.

**FIGURE 7:**
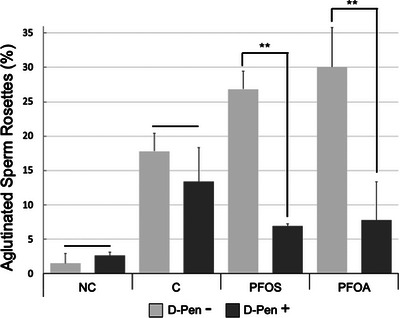
Effect of PFOS and PFOA on spermatozoa agglutination G1 during capacitation. Spermatozoa with G1 type agglutination (rosettes). NC, non‐capacitated; C, capacitated; D‐Pen, D‐penicillamine. Mean ± SD, *p* < 0.05. **Significant differences between groups; *n* = 5 in duplicate.

Sublethal doses were used to investigate the mechanism through which PFOS and PFOA induced agglutination during capacitation of the boar spermatozoa analysed. After 4 h of capacitation, the spermatozoa samples were classified by direct observation under a phase contrast microscope, observing that the NC group had mostly free spermatozoa (G0 = 97%). In the Cap control group, there was a significant increase of agglutinate rosette‐type agglutinates classified as G1 (18%) compared to the NC group (2.5%) (Figures 6 and 7). Another significant observation was that when the samples were exposed to PFOS and PFOA during capacitation (Cap + PFOS and Cap + PFOA), several rosettes (Type G1 agglutinates) bonded together to form large, motionless (only vibrant) aggregates of these rosettes, with 27% for PFOS and 30% for PFOA. This finding revealed a significant difference to the control group (Cap) (Figures 6 and 7). G2 and G3 agglutinates were observed in only small amounts in all groups (< 3%), so no significant differences appeared, and for this reason, they were not shown in this work  .

### 3.5 d‐Pen Prevented the Formation of Small Rosettes Caused by PFOS and PFOA During Capacitation

The NC and NC + d‐Pen control groups presented < 2.5% of rosette‐like agglutinations. No significant difference was observed between the control group Cap (18%) and the Cap + d‐Pen (13%) of rosette‐like agglutination (Type G1). These data indicated that d‐Pen did not affect preventing rosette agglutination in the NC and Cap groups. However, in the groups of spermatozoa exposed to PFOS and PFOA, adding d‐Pen during capacitation resulted in a significantly lower proportion of rosettes (Type G1), with only 7% in both cases (Figure 7). These results suggest that the d‐Pen prevents rosette formation (Type G1). Also, spermatozoa with d‐Pen have similar motility to that of the control group Cap.

### 3.6 d‐Pen Increased the Presence of Free SH Groups in Cap Spermatozoa Exposed to PFOS and PFOA

**FIGURE 8:**
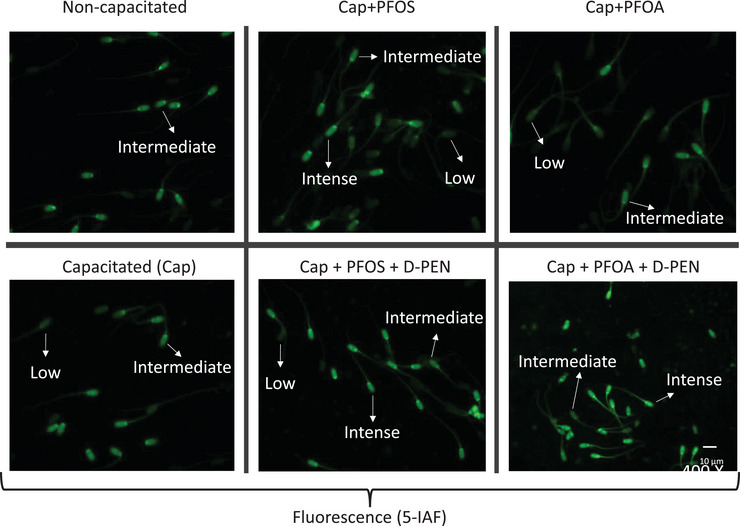
Effect of PFOS and PFOA on SH groups. Representative 5‐IAF fluorescence fields of non‐capacitated and capacitated spermatozoa exposed to PFOS and PFOA, with and without D‐Pen. 5‐IAF fluorescence was classified as low, intermediate and intense, indicating a lower or higher amount of SH groups on the surface of the spermatozoa. The intermediate and intense fluorescence values were considered indicators of the presence of SH groups.

In the Cap group, intermediate fluorescence was observed in 80% of the spermatozoa, significantly higher than in the NC group. Low fluorescence predominated in the PFOS‐treated spermatozoa at 67%, with intermediate fluorescence at only 34%. This was significantly different from the Cap group. Intermediate fluorescence was 71% when d‐Pen was added to the PFOS‐treated spermatozoa (PFOS + d‐Pen), producing a significant difference from the PFOS group. In the spermatozoa exposed to PFOA, 65% showed low fluorescence, while intermediate fluorescence was observed in only 26%. Once again, this result was significantly different from the Cap group. When d‐Pen was added (PFOA + d‐Pen), 50% of the spermatozoa showed intermediate fluorescence, while 50% had intense fluorescence, marking a significant difference from the PFOA group. The NC group had fewer SH groups on the surface of the spermatozoa, while the Cap group had a significantly higher amount. PFOS and PFOA reduced the number of SH groups and correlated with an increase in the number of agglutinated rosettes. Adding d‐Pen increased the number of SH groups in the spermatozoa exposed to PFOS and PFOA (Figure 8). These results reveal the role of d‐Pen in preventing rosette‐type agglutinations due to its effect in reducing the disulphide bonds that are responsible for PFOS or PFOA‐induced sperm–sperm agglutination (Table 1).

**TABLE 1:** 5‐IAF fluorescence equivalent to the number of SH groups of spermatozoa treated with PFOS, PFOA and D‐Pen.

Fluorescence (A.F.U.)	NC	C	C + PFOS	C + PFOS + DPEN	C + PFOA	C + PFOA + DPEN
Low	31 ± 5.50^a^	14 ± 3.22^a^	**61 ± 2.00** ^a^	27 ± 10.0^a^	**65 ± 2.21** ^a^	2 ± 1.00^a^
Intermedia	**65 ± 4.56** ^a^	**80 ± 4.04** ^a^	34 ± 2.83^a^	**70 ± 0.34** ^a^	26 ± 3.30^a^	48 ± 1.55^a^
Intense	3 ± 0.52	6 ± 0.82	5 ± 0.72	3 ± 1.23	9 ± 2.62	**50 ± 0.77** ^a^

*Note*: The values are presened as mean ± SD. *n* = 5 in duplicate.

Abbreviation: A.F.U., arbitrary fluorescence units.

^a^ A statistical difference between groups compared to the capacitated control group (*p* < 0.05).

## 4 Discussion

In this study, we observed that high PFOS and PFOA concentrations lead to larger agglutination of spermatozoa (G2 and G3). At sublethal concentrations, these chemicals formed smaller, rosette‐like agglutinates (G1). A lack of progressive motility characterised both types of agglutinates as only vibrant. The lack of motility in the spermatozoa within the agglutinates is likely due to the physical constraints imposed by aggregation, which prevented effective movement. It is well established that for spermatozoa to fertilise an egg, they must be free from any form of conjugation or agglutination (Monclus and Fornes 2016). It is important to note that PFAS can perturb the blood–testis barrier (Wan et al. 2014). PFAS are called ‘forever substances’ due to their physico‐chemical characteristics. There are damage indicator factors that reveal that PFAS reach spermatozoa in the testis, epididymis and oviduct of the female, potentially causing reproductive damage in the testis, causing damage to spermatogenesis, steroidogenesis and spermatozoa quality (Kim et al. 2020; Ma et al. 2023; Zhang et al. 2014). Due to PFAS exposure, spermatozoa agglutination can decrease spermatozoa quality and adversely affect fertilisation. Spermatozoa agglutination limits motility, a crucial factor for successful fertilisation (World Health Organisation 2021). Wild boars exhibit remarkably high PFAS concentrations due to the extensive exposure and slow elimination of half‐lives (Rupp et al. 2023). For example, there were found ≤ 1780 and ≤ 28.6 µg/kg of PFOS in liver and muscle samples of domestic and wild boars (Oseguera‐Lopez et al. 2020). The prevalence of PFAS in the environment is underscored by the findings of Guruge et al. (2008), suggesting that those who reported species‐specific concentrations of perfluoroalkyl contaminants in farm and pet animals in Japan, their research highlights the widespread occurrence of these contaminants in agricultural settings, raising concerns about their potential impact on livestock health and reproductive performance. Understanding the implications of these pollutants on boar sperm physiology can inform breeding practices and reproductive technologies aimed at enhancing fertility outcomes (Raymer et al. 2012).

We observed that boar spermatozoa can build agglutinates Type G1, corresponding to rosettes joined head–head; upon capacitation conditions, these rosettes cooperate to progressive movement. Upon exposing Cap spermatozoa to sublethal concentrations of PFOS and PFOA, we observed a significant decrease in the number of spermatozoa that achieved capacitation. In addition, the formation of G1‐type agglutinates—a characteristic of physiological rosettes associated with spermatozoa capacitation—increased significantly, and those rosettes formed immobile aggregates. Specifically, agglutinate formation increased approximately 1.5‐fold with PFOS and 2‐fold with PFOA compared to the control group of Cap spermatozoa. At higher PFAS concentrations (1 and 1.5 mM), larger agglutinates were observed with heterogeneous interactions, including head–head, head–flagellum and combinations.

In contrast, at low sublethal concentrations, the agglutinates formed primarily specific head–head interactions that resulted in rosettes that aggregated further. Those aggregates may not have occurred due to specific interactions but were influenced by components of the cellular microenvironment, possibly due to the PFAS. Some authors have proposed that rosettes may result from physiological effects associated with spermatozoa capacitation. In the control group Cap, a typical, balanced formation of rosettes was observed, characteristic of spermatozoa capacitation (Harayama et al. 1998; Salgado‐Lucio et al. 2020). However, in the presence of PFAS, we noted an excess and imbalance of rosette formation that could impair fertilisation by reducing the availability of free spermatozoa to reach the egg.

PFOS and PFOA have been associated with decreased spermatozoa quality, as males with detectable levels of PFOS and PFOA in their blood exhibit reduced spermatozoa motility and other key quality parameters (Šabović et al. 2020; Wan et al. 2014). In previous work by our group, we observed that PFOS and PFOA caused membrane damage that prevented the release of cholesterol during capacitation (Ortiz‐Sanchez et al. 2022). We further found that these substances increased cholesterol levels and disrupted the proper reorganisation of essential molecules, including membrane microdomains and glycocomponents crucial for the capacitation process (Ortiz‐Sanchez et al. 2022).

The formation of rosettes in boar spermatozoa and the mechanisms behind their formation have yet to be extensively studied. It has been hypothesised that changes in, and damage to, the plasma membrane caused by toxicants like PFAS may lead to nonspecific interactions between spermatozoa that promote the formation of non‐mobile agglutinated rosettes. Moreover, these toxicants, or their products, such as reactive oxygen species (ROS), might exert oxidative effects on thiol groups in plasma membrane proteins. Previous analyses by our group observed that PFAS could increase ROS levels during spermatozoa capacitation, causing damage that hinders this process (Oseguera‐Lopez et al. 2020).

Based on this hypothesis, we employed d‐Pen, following the work of Leahy et al. (2016), who elucidated the copper‐induced agglutination mechanism. They demonstrated that oxidation of SH thiol groups by copper leads to the formation of disulphide bonds on the surface of ram spermatozoa, resulting in agglutination. d‐Pen was shown to reduce these bonds, making the agglutination reversible (Leahy et al. 2016). d‐Pen, a penicillin‐derived drug used to treat Wilson's disease, acts as a copper chelator without antibiotic properties. In Wilson's disease, copper is not eliminated but accumulates and becomes harmful.

In our study, the control group showed no changes in rosette formation with or without d‐Pen, indicating that this substance does not affect the physiological formation of rosettes. However, in the presence of d‐Pen, the spermatozoa exposed to PFOS and PFOA exhibited fewer rosettes and a higher proportion of free spermatozoa than those not treated with d‐Pen. This finding supports the assertion that d‐Pen prevents rosette formation caused by PFAS.

Leahy et al. (2016) observed that the deagglutination effect of d‐Pen is due to its properties as a reducing agent due to the presence of a thiol group. They noted that neither a potent copper chelator like bathocuproinedisulphonic acid disodium salt nor a broad‐scale chelator like EDTA produced a similar effect. Likewise, Talevi et al. (2007) reported that reducing agents with thiol groups, such as d‐Pen, β‐mercaptoethanol and cysteine, participate in the release of spermatozoa that have adhered to the tubal epithelium of bulls in vitro. They suggested that redox regulation of thiol groups on the spermatozoa surface may influence spermatozoa adhesion to the female reproductive tract's epithelium, which could affect spermatozoa quality and fertilising capacity. Therefore, d‐Pen may reduce the disulphide bonds of the rosettes caused by PFAS due to its capacity as a reducing agent and the oxidation of its thiol group.

de Lamirande and Gagnon (2003) stated that spermatozoa capacitation is associated with an increase in free SH groups in triton‐soluble proteins. Their work showed more SH groups in Cap spermatozoa that were distributed homogeneously in the head, the midpiece and part of the flagellum. This distribution pattern is like that observed by Talevi et al. (2007) in 85% of free spermatozoa in bulls. Our study also found an increase in SH groups when spermatozoa were treated with d‐Pen in the presence of PFOS and PFOA, compared to treatment with either PFOS or PFOA alone, though the SH levels recorded did not reach those seen in fully Cap spermatozoa. Our results also showed that d‐Pen reduced the proportion of rosettes caused by PFOS and PFOA by approximately four times while increasing the number of free spermatozoa.

The increase in SH groups under d‐Pen treatment supports the hypothesis that the interaction between spermatozoa in rosettes in the presence of PFOS and PFOA occurs through disulphide bonds following the oxidation of SH groups. d‐Pen effectively reduced these disulphide bonds. Possible candidate proteins with free SH groups on the membrane surface are ADAM (a disintegrin and metalloproteinase), as inhibition of the metalloproteinase domain in a study by Leahy et al. (2016), which led to an increase in the number of non‐agglutinated spermatozoa and indicated their role in copper‐induced agglutination. Compared to their results, Leahy et al. (2016) also show that d‐Pen prevented physiological agglutination of capacitation in the ram spermatozoa in the TALP medium; we did not observe this effect in the boar. This is probably because our model can form fewer rosettes during capacitation than the ram. Furthermore, rosette formation in rams is slightly different from that in boars. Rosettes in rams are groups of spermatozoa joined head‐head but arranged in a unidirectional manner. It is interesting to investigate the mechanism of action of rosette formation during capacitation spermatozoa in boar.

We have observed that the excessive formation of rosettes in response to exposure to PFOS and PFOA reflects toxic damage that impairs spermatozoa motility and capacitation. This effect is likely due to redox deregulation caused by the generation of ROS by PFAS, which affects proteins with free SH groups, such as the ADAM family.

## 5 Conclusions

Boar spermatozoa form rosettes during capacitation. When spermatozoa were exposed to PFOS or PFOA, we found an increased formation of immobile rosette‐type agglutinates and aggregates; we demonstrated that d‐Pen prevents them because it led to a notable increase in free SH groups in spermatozoa exposed to PFOS or PFOA, which produced a reduction in disulphide bridges, that typically facilitate rosettes. These observations suggest that the agglutination mechanism induced by PFOS and PFOA likely operates through the oxidation of SH groups, forming disulphide bonds that promote spermatozoa agglutination. d‐Pen is a drug with low toxicity that could be used in animal‐assisted reproduction for spermatozoa agglutination infertility, reverting the oxidation caused by toxicants like PFAS.

## Author Contributions

**Paola Berenice Ortiz‐Sánchez**: investigation, methodology, writing–original draft, formal analysis, supervision. **Yolitzin A. Miranda‐Ruiz**: investigation, methodology. **Leslye Sámano‐Hernández**: formal analysis, writing–review and editing. **Irma Jiménez‐Morales**: formal analysis, writing–review and editing. **Humberto González‐Márquez**: conceptualisation, formal analysis, writing–review and editing. **Reyna Fierro**: conceptualisation, formal analysis, writing–review and editing, project administration and resources.

## Ethics Statement

The authors declare that semen samples were obtained using the glove‐hand method from a commercial boar stud, which complies with the health and animal welfare regulations of the government of Mexico, according to the Official Standard NOM‐062‐ZOO‐1999, which stipulates the regulations for the care and use of laboratory animals.

## Conflicts of Interest

The authors declare no conflicts of interest.

### Peer Review

The peer review history for this article is available at https://publons.com/publon/10.1002/vms3.70244.

## Data Availability

The data that support the findings of this study are available on request from the corresponding author.
